# Establishment of the HPLC fluorescence detection method for plasma trace ivermectin and its pharmacokinetics in Bactrian camel

**DOI:** 10.1002/vms3.1447

**Published:** 2024-04-12

**Authors:** Bin Hou, Haifeng Wang, Nan Jiang, Bilige Haosi, Surong Hasi

**Affiliations:** ^1^ Key Laboratory of Clinical Diagnosis and Treatment Technology in Animal Diseases, Ministry of Agriculture, College of Veterinary Medicine Inner Mongolia Agricultural University Hohhot China; ^2^ Inner Mongolia Tenghetai Camel Industry Co., Ltd Bayannur China; ^3^ Tongliao Institute of Agricultural and Animal Husbandry Sciences Tongliao China

**Keywords:** Bactrian camel, ivermectin injection, HPLC fluorescence detection, pharmacokinetic characteristics

## Abstract

**Background and objective:**

Ivermectin (IVM), a widely used veterinary anthelmintic, lacks recommended doses for Bactrian camels. This study aims to establish its pharmacokinetics in Bactrian camels, comparing with other livestock.

**Methods:**

A method for high‐performance liquid chromatography fluorescence detection of IVM in plasma was developed.

**Results:**

IVM exhibited linear scaling (*y* = 0.6946*x* + 0.0088, *R*2 = 0.9988) within 0.025–5 ng/mL, with a lower limit of quantification of 25.00 pg/mL, high recovery (>70%) and low RSD (<7%). In Bactrian camels, IVM injection showed a low *C*_max_, extended *T*_max_ and apparent secondary absorption compared to cattle and sheep.

**Conclusions:**

Slow absorption and widespread distribution were observed, with peak concentration and area under the curve correlating positively with the dose. This study provides insights into IVM pharmacokinetics in Bactrian camels, informing dose determination and highlighting potential metabolic differences compared to other livestock.

## Graphical abstract

The main pharmacokinetic parameters of ivermectin injection in Bactrian camels had a low *C*_max_, a long *T*_max_ and obvious secondary absorption phenomenon compared to cattle and sheep. In addition, ivermectin is slowly absorbed and widely distributed in the Bactrian camel, and its peak concentration and area under the concentration–time curve are positively correlated with the dose administered.

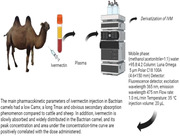

## 1 INTRODUCTION

Ivermectin (IVM) is a derivative of avermectin B1's catalytic hydrogenation process (Armour et al., 1980). Initially, IVM was introduced to the market as an anthelmintic in France during the early 1980s, which later, in 1987, experienced its first use as an anthelmintic for humans. Presently, the compound is deployed extensively in clinical practices due to its highly efficient anthelmintic action, showing substantial efficacy when dealing with internal and external parasites, such as mites, ticks, lungworms and gastrointestinal nematodes, in a variety of animal species (Claerebout et al., 1997; Mckellar & Gokbulut, 2012). Furthermore, IVM demonstrates a broad range of anthelmintic activities, high efficacy, low residual effect and a safe profile, thus adding to its popularity as a veterinary anthelmintic (Van Hees et al., 2020).

IVM attaches to the inhibitory neurotransmitter γ‐aminobutyric acid (γ‐GABA) and its receptor pathway (Casida & Durkin, 2015), eliciting the release of copious amounts of γ‐GABA from the parasites. This leads to their demise through tardive dyskinesia (Campbell, 2012). Interestingly, IVM is remarkably safe for mammals, as their principal peripheral neurotransmitter is acetylcholine (Vercruysse et al., 2008).

Bactrian camels, Camelo of the family Camelidae, Artiodactyla, display unique biological characteristics like resistance to heat, cold, drought and thirst. These characteristics favour the survival of these ‘ships of the desert’ in arid, partially arid conditions (Hasi et al., 2018). Plentiful in the Taklamakan Desert, Lop Nor, along the border of China and Mongolia, and the uninhabited areas of the northern Altunshan Mountain, the numbers of wild Bactrian camels nevertheless are in decline, prompting their inclusion in the IUCN Red List of Threatened Species.

Given the economic value derived from Bactrian camels through their milk, fleece and meat, camel farming represents an important source of income for herders in these regions. Bactrian camels also add to the world's important species resources (Burger et al., 2019; Ji et al., 2009). The semi‐wild grazing behaviour they typically display can present significant challenges when it comes to parasite prevention and control (Al Anazi and Alyousif, 2011; Ballweber, 2009). Such parasitic infections can interfere with the growth and development of Bactrian camels, impacting the quantity and quality of their milk, meat and fleece output. Certain parasites can even cause zoonotic diseases, representing a significant threat to public health (Hamidinejat et al., 2013). Elaborating on this, when Martin et al. (2021) reported resistance to IVM, the frequency of IVM use in Bactrian camels was found to be low. Therefore, IVM still represents an effective anthelmintic for these animals (Van Straten & Jongejan, 1993).

Pharmacokinetic studies of IVM have been conducted in sheep, cattle, pigs, rabbits and rats (González Canga et al., 2009; Lifschitz et al., 2004; Molento et al., 2004; Vercruysse et al., 2008), providing a comprehensive database, but no such studies have been conducted in Bactrian camels, hampering clinical applications for suitable therapeutics. As a result, this study aims to investigate their pharmacokinetic parameters, providing a scientific basis for future clinical use of IVM in Bactrian camels.

## 2 MATERIALS AND METHODS

### 2.1 Animal grouping and blood sample collection

Twelve camels (average weight 420.42 ± 51.9 kg) were divided into two groups of six; one group received a low IVM dose (0.2 mg/kg) and the other a high dose (0.3 mg/kg). The IVM injection used in the experiment was provided by Inner Mongolia Huao Kexing Biotechnology Co., Ltd., with a production batch number of 050051128. According to high‐performance liquid chromatography (HPLC) testing, its content was found to be 103% of the labelled content. Drugs were administered via a subcutaneous injection in the neck, proportionate to the weight of the camel, followed by a blood sample collection from the jugular vein. Blood samples, approximately 5 mL, were collected into heparin sodium blood collection tubes, mixed and then centrifuged at 4000 r/min for 10 min. Supernatants obtained were stored in a refrigerator at −20°C for future use. Blood collection timings were as follows: pre‐medication (0 h), and 1 h, 3 h, 7 h, 12 h, 1–3‐, 5‐, 9‐, 15‐, 23‐, 33‐, 45‐ and 60‐day post‐medication.

### 2.2 Development of HPLC fluorescence detection technique for camel plasma samples and methodology verification

#### 2.2.1 Formulation of standard stock solutions

Doramectin (DOR) standard was purchased from the Chinese Veterinary Medicine Standard Substance Ordering Platform, with a purity of 95.0%. Formulation of the IVM (DOR) standard reserve solution involves weighing precisely 1 mg of IVM (DOR) standard, diluting it 10‐fold using chromatographic grade methanol in a 10 mL brown volumetric flask to create a 100 µg/mL standard reserve solution. This solution is then further diluted 10‐fold to yield a 10 µg/mL standard reserve solution. This is stored at −20°C in a sealed, light‐protected environment. For the preparation of the IVM standard working solution, allow 10 µg/mL of the reserve solution to acclimate to room temperature before diluting to concentrations of 1000, 100 and 10 ng/mL before storing at −4°C. Similarly, for the preparation of the DOR standard working solution, bring 10 µg/mL of the stock solution to room temperature and dilute it to a concentration of 20 ng/mL, followed by storage at −4°C.

#### 2.2.2 Plasma sample pre‐treatment

The plasma samples were thawed at room temperature and directly added to solid‐phase extraction columns for drug enrichment. The solid‐phase extraction columns were pre‐activated by soaking in anhydrous methanol for 12 h. Before usage, they were rinsed with 2.5 mL of methanol, followed by 2.5 mL of ultrapure water. When the water surface reached approximately 0.1 cm from the packing surface, the control valve was closed to begin sample loading. Initially, a 20.0 ng/mL DOR standard working solution was taken, and 50 µL was accurately pipetted into the solid‐phase extraction column using a micropipette. Then, 1.00 mL of plasma sample was precisely aspirated and added to the solid‐phase extraction column. After sample addition, the control valve was opened to allow the sample to flow naturally. Once the sample had flowed out, it was rinsed with 7.5 mL of ultrapure water, followed by 7.5 mL of 25% methanol. Subsequently, the solid‐phase extraction column was dried, and after complete drying, it was eluted with 5 mL of anhydrous methanol. The eluate was collected in a 10.0 mL centrifuge tube and dried under nitrogen gas at 50°C before fluorescence derivatization. Additionally, during sample processing each day, quality control samples with concentrations of 2, 0.30 and 0.075 ng/mL were prepared. The quality control samples underwent preprocessing using the same method as the plasma samples for each batch of plasma samples.

#### 2.2.3 Derivatization of IVM

The derivatization involves using two reagents, with Derivatization Reagent A being a 1:2 blend of trifluoroacetic anhydride and acetonitrile and Derivatization Reagent B being a 1:1 mix of *N*‐methylimidazole and Acetonitrile. The derivatization process involves a sequence of steps in which each reagent is added to the sample, thoroughly mixed and then allowed to react for a specified period.

#### 2.2.4 HPLC conditions

The HPLC conditions would include specific parameters, such as the mobile phase composition, the type of column, the detector settings, flow rate, temperature and the injection volume. Mobile phase: (methanol:acetonitrile = 1:1): water = 95.8:4.2. Column: Luna Omega 5 µm Polar C18 100A (4.6 × 150 mm^2^). Detector: fluorescence detector, excitation wavelength 365 nm, emission wavelength 475 nm. Flow rate: 1.0 mL/min. Temperature: 35°C. Injection volume: 20 µL.

#### 2.2.5 Construction of the standard curve

Take out the 1000 ng/mL IVM standard working solution, and prepare plasma sample standard curve solutions with concentrations of 0.025, 0.05, 0.10, 0.25, 0.50, 1.0, 2.5 and 5.0 ng/mL, respectively. Plasma samples undergo preprocessing and derivatization before HPLC detection. The ratio of the peak area of IVM to the internal standard DOR peak area is calculated. Plot the standard curve with IVM concentration as the *x*‐axis and the ratio of peak areas as the *y*‐axis.

#### 2.2.6 Tests for recovery, precision and minimum limits of detection and quantification

These tests involve preparing standard working solutions and blank plasma at various concentrations, carrying out tests and then calculating the absolute, extraction and spiked recoveries. Precision is established by preparing IVM quality control samples at various concentrations and batches. Determination of the lowest detection limit (LOD) and quantification limit (LOQ): Blank plasma samples were taken, and appropriate amounts of IVM standard stock solutions were added to prepare plasma samples with concentrations of 0.01, 0.025, 0.05 and 0.1 ng/mL, respectively. Sample processing was carried out following the methods outlined in Sections 2.2.3.2 and 2.2.3.3, and the detection analysis was conducted according to the HPLC conditions described in Section 2.2.3.4. The plasma sample drug concentration was considered the LOD when the signal‐to‐noise ratio S/N was ≥3, and it was considered the LOQ when S/N was ≥10.

### 2.3 Pharmacokinetic characterization of IVM injection in Bactrian camels

IVM blood concentration in the plasma samples at each blood collection point is calculated based on the standard curve of IVM in camel plasma under the set HPLC conditions. The blood concentration–time data is analysed using the Phoenix WinNonlin 7.00 pharmacokinetics analysis software based on a non‐compartmental model to determine the pharmacokinetic parameters of IVM in camel blood.

## 3 RESULTS

### 3.1 The establishment of a method for determining IVM blood concentration in Bactrian camel plasma

#### 3.1.1 Method specificity

**FIGURE 1:**
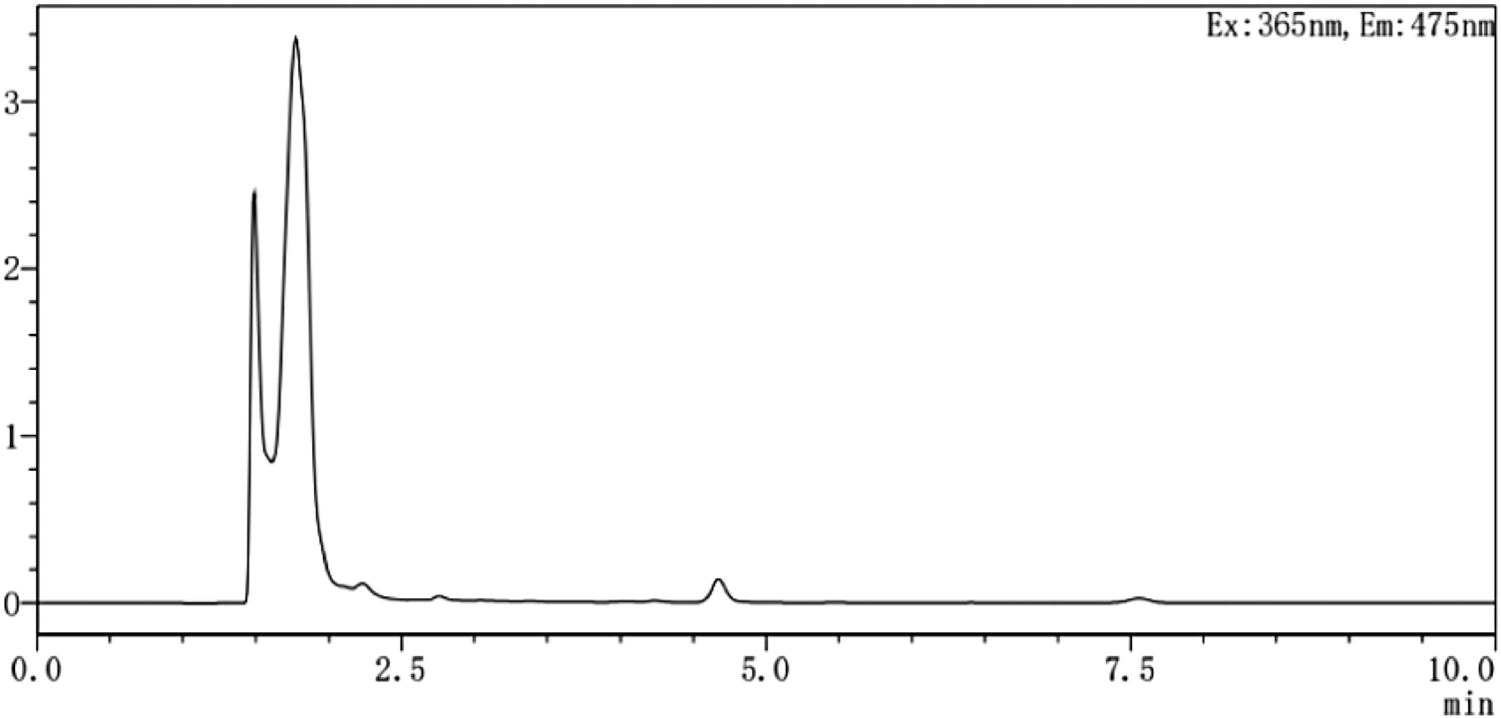
Chromatogram of control plasma.

**FIGURE 2:**
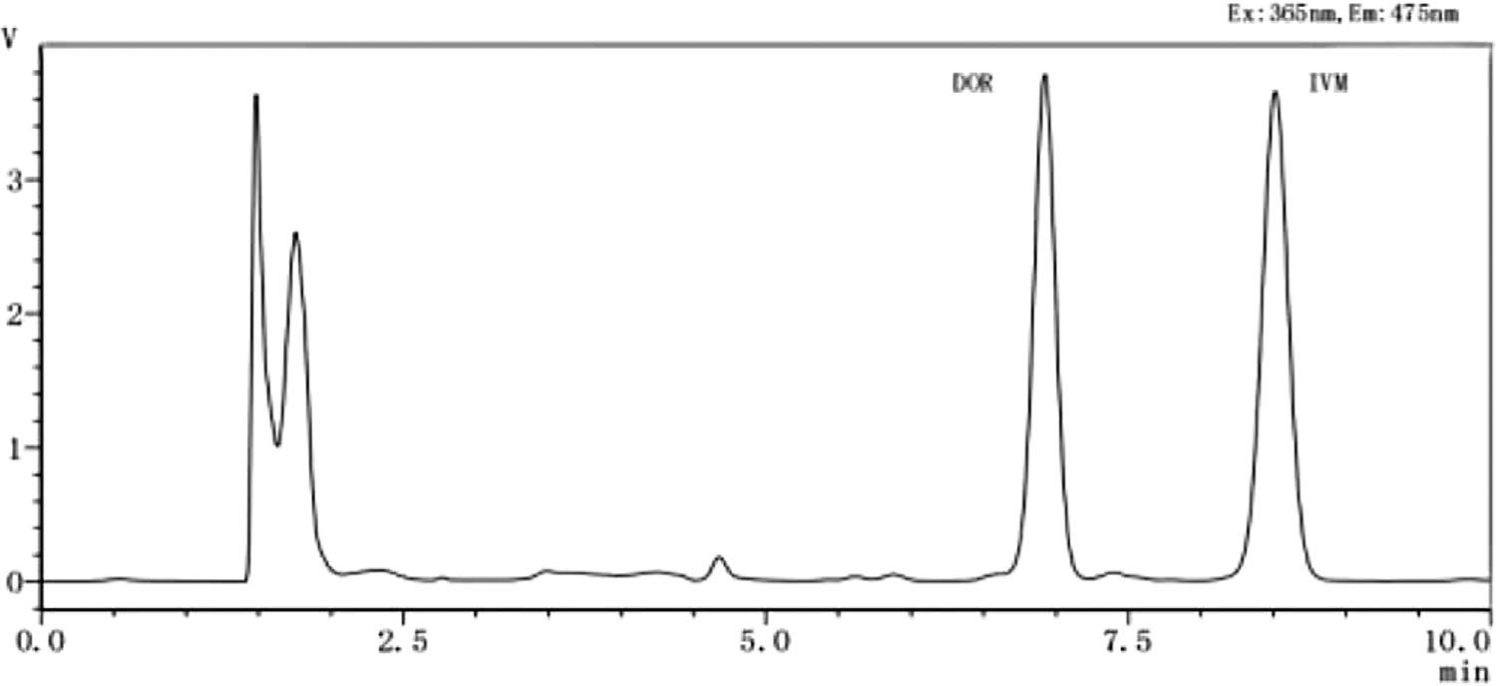
Chromatogram of doramectin (DOR) and ivermectin (IVM) standards.

**FIGURE 3:**
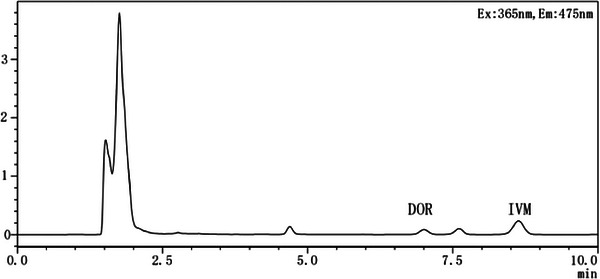
Chromatogram of the Bactrian camel plasma samples.

The chromatograms of the Bactrian camel control plasma, DOR and IVM standard control plasma, and the Bactrian camel plasma samples can be observed in Figures 1, 2, 3.

#### 3.1.2 Standard curve and limit of quantification results

**FIGURE 4:**
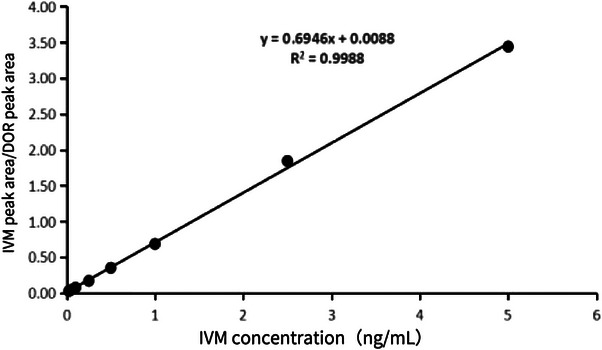
Standard curve diagram of ivermectin (IVM) concentration‐IVM peak area/doramectin (DOR) peak area.

The ratio of the IVM peak area to the DOR peak area vs. IVM concentration provided a good linear regression within the concentration range of 0.025–5 ng/mL. The linear regression equation was *y* = 0.6946*x* + 0.0088, with *R*2 = 0.9988, and the results of this linear regression are revealed in Figure 4. The lowest LOD and the LOQ were 8.00 and 25.00 pg/mL, respectively, indicating that the method has sufficient sensitivity to meet the requirements of drug detection.

#### 3.1.3 Recovery test results

Tables 1, 2, 3 depict the results for absolute recovery, extraction recovery and spiked recovery of IVM, respectively. These results are consistent with the needs of a pharmacokinetic test.

**TABLE 1:** Ivermectin (IVM) absolute recovery results.

Sample concentration (ng/mL)	IVM absolute recovery rate			Mean ± SD	RSD (%)
	1	2	3		
0.08	72.50	70.00	73.75	72.08 ± 1.91	2.65
0.30	75.99	73.00	74.00	74.00 ± 1.00	1.35
0.50	85.60	83.60	82.80	84.00 ± 1.44	1.72

**TABLE 2:** Ivermectin (IVM) extraction recovery results.

Sample concentration (ng/mL)	IVM absolute recovery rate			Mean ± SD	RSD (%)
	1	2	3		
0.08	101.72	100.00	96.55	99.43 ± 2.63	2.15
0.30	89.02	91.46	90.24	90.24 ± 1.22	1.31
0.50	110.88	108.29	107.25	108.81 ± 1.87	1.54

**TABLE 3:** Ivermectin (IVM) plus sample recovery results.

Sample concentration (ng/mL)	IVM absolute recovery rate			Mean ± SD	RSD (%)
	1	2	3		
0.08	79.58	70.83	74.58	75.00 ± 4.39	5.85
0.30	76.22	74.56	72.22	74.33 ± 2.01	2.70
0.50	86.73	85.53	97.13	89.80 ± 6.38	7.10

#### 3.1.4 Results for precision test, LOD, and LOQ

The LOD and LOQ values (8.00 and 25.00 pg/mL, respectively) endorse the fact that this method is highly sensitive and fulfils the demands of drug detection.

### 3.2 Pharmacokinetic characterization of IVM results

#### 3.2.1 Blood concentration determination of IVM in Bactrian camel plasma

**FIGURE 5:**
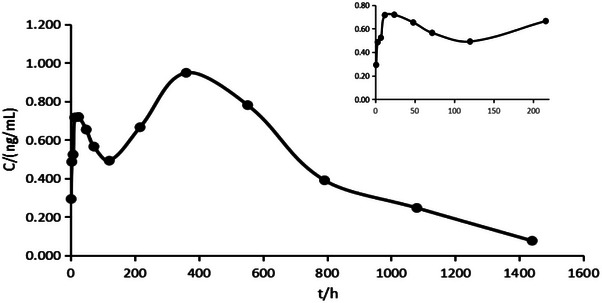
Mean (*n* = 6) plasma concentrations of ivermectin (IVM) low‐dose group (0.2 mg/kg) after subcutaneous administration to Bactrian camel.

**FIGURE 6:**
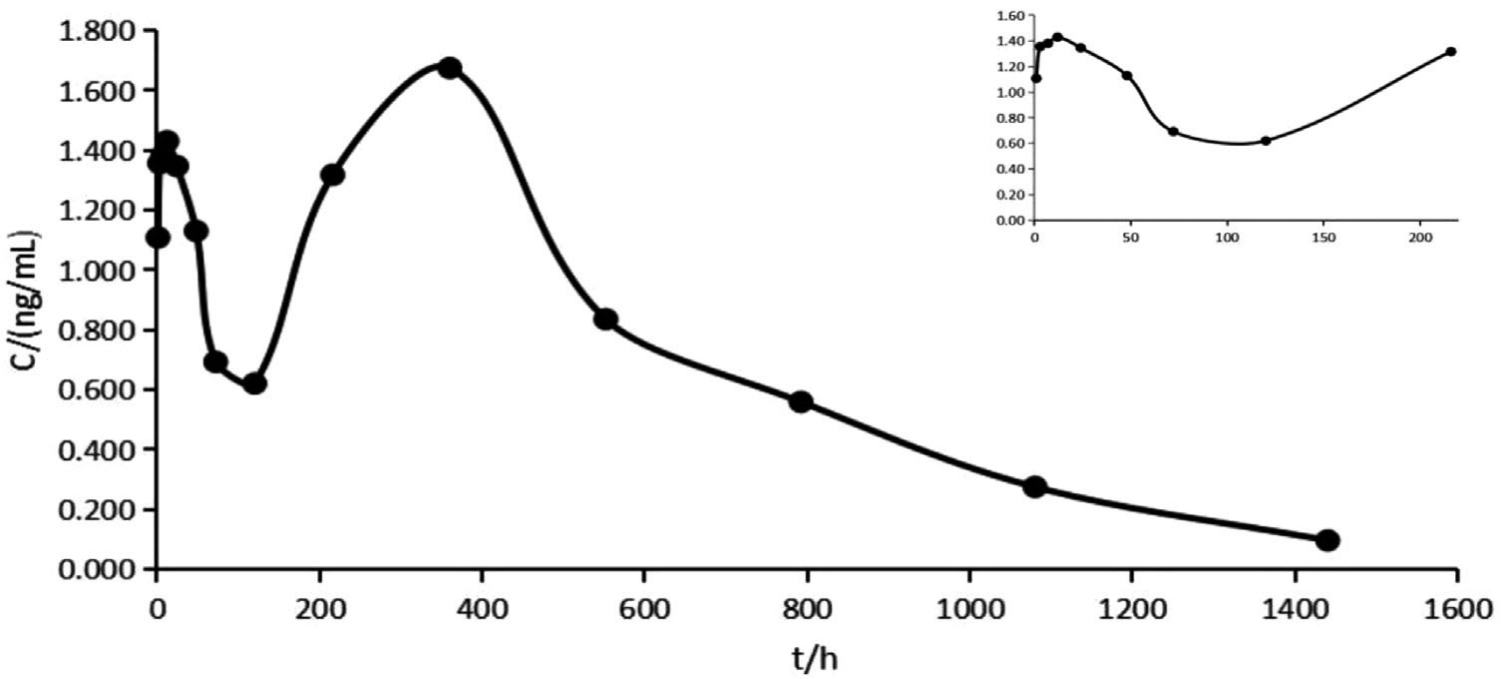
Mean (*n* = 6) plasma concentrations of ivermectin (IVM) high‐dose group (0.3 mg/kg) after subcutaneous administration to Bactrian camel.

Following the test method detailed in Section 2.2, Bactrian camels were given single, subcutaneous doses of 0.2 and 0.3 mg/kg. Blood was collected at defined time points, and the average blood concentration–time curves are presented in Figures 5 and 6.

#### 3.2.2 Pharmacokinetic data

The blood concentration values of IVM at each time point were analysed using the Phoenix WinNolin 7.0 pharmacokinetic analysis software, following a non‐compartmental model approach. The primary pharmacokinetic parameters of IVM in Bactrian camel blood are disclosed in Tables 4 and 5.

**TABLE 4:** Ivermectin (IVM) plus sample recovery results.

Theoretical concentration (ng/mL)	IVM intra‐batch precision		IVM lot‐to‐lot precision	
	Measured value (%)	RSD (%)	Measured value (%)	RSD (%)
0.08	0.067 ± 0.002	2.460	0.067 ± 0.002	3.416
0.30	0.271 ± 0.002	0.718	0.272 ± 0.003	1.265
0.50	0.467 ± 0.021	1.127	0.451 ± 0.024	1.317

**TABLE 5:** Major pharmacokinetics parameters in the ivermectin (IVM) low‐ and high‐dose groups.

Parameter	Unit	Parameter value of low‐dose (*x* ± SD)	Parameter value of high‐dose (*x* ± SD)
*T*_max_	Day	12.20 ± 6.26	12.84 ± 7.69
*C*_max_	ng/mL	1.08 ± 0.32	1.71 ± 0.26
AUC	Day·ng/mL	29.09 ± 7.12	42.44 ± 12.14
MRT	Day	20.69 ± 2.66	20.60 ± 2.36
*T*1/2	Day	11.02 ± 2.68	9.97 ± 2.19
CL	mL/h/kg	284.88 ± 70.76	301.65 ± 83.04
Vd	L/kg	115.95 ± 73.59	113.52 ± 49.53

Abbreviation: AUC, area under the curve; CL, clearance; MRT, mean residence time.

## 4 DISCUSSION

Bactrian camels often experience intense parasitic infections such as tick bites, vaginal myiasis and gastrointestinal nematodes (e.g. *Trichostrongylus*, *Nematodirus* and *Parabronema skrjabini*). Gastrointestinal nematodes, in particular, can result in anaemia, wasting, diarrhoea and even fatality. As Bactrian camels are usually semi‐wild grazers, they have an increased susceptibility to such parasitic diseases. Despite their robust resistance, camel herders may often overlook these parasitic infections during the rearing process. IVM, with its broad‐spectrum, high efficacy, low toxicity and user‐friendliness, stands as one of the most effective antiparasitic drugs worldwide. However, to date, no pharmacokinetic studies of IVM in Bactrian camels have been reported, leading to a lack of scientific basis for clinical application.

IVM detection methods are highly diversified, including such techniques as liquid chromatography–ultraviolet detection, liquid chromatography–fluorescence detection, liquid–liquid–mass spectrometry, thin‐layer chromatography and immunoassays, with the first three being the most commonly utilized. Fluorescence detection has gained widespread use due to its heightened sensitivity for quantitative detection, thereby facilitating the detection of IVM residues at extremely low concentrations. The fluorescence derivatization reaction is a critical step in this experiment (Oukessou et al., 1999).

The design of the study was based on prior research, and the blood collection points were set to cover the absorption, distribution and elimination phases. This approach was guided by existing literature which encouraged a minimum of three blood collection points for each phase. From the drug–time profile, it was clear that the pharmacokinetic parameters of IVM were significantly different in Bactrian camels compared to cattle and sheep (Lifschitz et al., 1999, 2004; Perez, 2002; Gonzalez et al., 2007; Ali et al., 1996; Badri et al., 1996).

A non‐atrial compartment model was used to interpret the blood concentration–time data, and several significant differences in pharmacokinetic parameters were found compared to cattle and sheep. For example, the peak plasma concentration of IVM in Bactrian camel was notably lower, whereas the time to reach peak concentration was extended. Although the peak concentration and area under the curve (AUC) of the high‐dose group were approximately 1.5 times higher than those of the low‐dose group, no other significant differences in pharmacokinetic parameters were observed.

According to the research of Oukessoua M, the pharmacokinetic parameters of IVM in the blood plasma of camels after subcutaneous injection of a dose of 0.2 mg/kg were significantly different from those of other ruminants such as cattle and sheep, but they were similar to those of cattle and sheep in this experiment. The *C*_max_ value of IVM in the blood plasma of lactating camels was significantly lower than that in the blood plasma of cattle and sheep, whereas the *T*_max_ value in the blood plasma of camels was lower than that in the blood plasma of cattle but higher than that in the blood plasma of sheep. The *C*_max_ value of IVM in the blood plasma of lactating camels was lower than that in the blood plasma of cattle but significantly higher than that in the blood plasma of sheep, whereas the *T*_max_ value of camels was lower than that in the blood plasma of cattle but significantly higher than that in the blood plasma of sheep. The MRT value of IVM in the blood plasma of lactating camels was similar to that in the blood plasma of cattle but significantly higher than that in the blood plasma of sheep. The AUC value of IVM in the blood plasma of lactating camels was significantly lower than that in the blood plasma of cattle and sheep. The reason for the lower concentration of IVM in the blood plasma of camels may be that the drug is chelated in camel fat or highly bound to plasma proteins. The above pharmacokinetic results may indicate that subcutaneous injection of 0.2 mg/kg of IVM in camels is less effective than in other ruminants. However, this dose is clinically effective against most of the ectoparasites and endoparasites in the camels. It is possible that the parasites in camels may be exposed to low concentrations of IVM for a long time, as subdivided doses of IVM can increase the efficacy of anthelmintics. However, long‐term use of low concentrations of drugs is more likely to produce resistance in worms, and camels are not often dewormed; there are few reports of resistance to dewormers in camels, and there may be no such reports in current research. Therefore, the routine medication of camels should not be the same as that of cattle and horses. The withdrawal period of camels is also different from that of ordinary animals, and medication should be given according to their characteristics to achieve good efficacy.

To sum up, we successfully established an HPLC–fluorescence detection method to determine the plasma concentration of IVM in Bactrian camels. Our research studies the pharmacokinetic characteristics of this drug, providing a scientific basis for rational clinical use of IVM in these animals.

## AUTHOR CONTRIBUTIONS

Investigation; writing – original draft; visualization; software: Bin Hou. Validation; writing – original draft: Haifeng wang. Investigation; visualization: Nan Jiang. Investigation; visualization, software: Bilige Haosi. Writing – review and editing; conceptualization; supervision; funding acquisition: Surong Hasi. All authors read and approved the final manuscript.

## CONFLICT OF INTEREST STATEMENT

The authors declare that the research was conducted in the absence of any commercial or financial relationships that could be construed as potential conflicts of interest.

### ETHICS STATEMENT

Animal procedures were performed in accordance with the National Standard Guideline for Ethical Review of Animal Welfare (GB/T 35892‐2018) and approved by the Animal Care and Use Committee of Inner Mongolia Agricultural University.

### PEER REVIEW

The peer review history for this article is available at https://publons.com/publon/10.1002/vms3.1447.

## Data Availability

The datasets generated during and/or analysed during the current study are not publicly available due to [Patent Application] but are available from the corresponding author on reasonable request.
